# Conditioned medium-preconditioned EPCs enhanced the ability in oligovascular repair in cerebral ischemia neonatal rats

**DOI:** 10.1186/s13287-021-02157-4

**Published:** 2021-02-12

**Authors:** Ning Zhou, Lei Wang, Ping Fu, Zihao Cui, Yuhang Ge, Feiyu Jiang, Jing Liu, Chao Ren, Zuo Luan, Hongbin Fan, Ruiqin Yao

**Affiliations:** 1 Department of Cell Biology and Neurobiology, Xuzhou Key Laboratory of Neurobiology, Xuzhou Medical University, Xuzhou, 221009 China; 2 Department of Human Anatomy, Xuzhou Medical University, Xuzhou, 221009 China; 3 Department of Neurology, Affiliated Hospital of Xuzhou Medical University, Xuzhou, 221002 China; 4 Department of Neurology, Affiliated Yantai Yuhuangding Hospital of Qingdao University, Yantai, 264000 China; 5 Pediatrics, the Sixth Medical Center of PLA General Hospital, Beijing, 100048 China

**Keywords:** Endothelial progenitor cells, Neonatal ischemia, Cytokine, White matter disease, Oligodendrocyte precursor cells

## Abstract

**Background:**

Oligovascular niche mediates interactions between cerebral endothelial cells and oligodendrocyte precursor cells (OPCs). Disruption of OPC-endothelium trophic coupling may aggravate the progress of cerebral white matter injury (WMI) because endothelial cells could not provide sufficient support under diseased conditions. Endothelial progenitor cells (EPCs) have been reported to ameliorate WMI in the adult brain by boosting oligovascular remodeling. It is necessary to clarify the role of the conditioned medium from hypoxic endothelial cells preconditioned EPCs (EC-pEPCs) in WMI since EPCs usually were recruited and play important roles under blood-brain barrier disruption. Here, we investigated the effects of EC-pEPCs on oligovascular remodeling in a neonatal rat model of WMI.

**Methods:**

In vitro, OPC apoptosis induced by the conditioned medium from oxygen-glucose deprivation-injured brain microvascular endothelial cells (OGD-EC-CM) was analyzed by TUNEL and FACS. The effects of EPCs on EC damage and the expression of cytomokine C-X-C motif ligand 12 (CXCL12) were examined by western blot and FACS. The effect of the CM from EC-pEPCs against OPC apoptosis was also verified by western blot and silencing RNA. In vivo, P3 rat pups were subjected to right common carotid artery ligation and hypoxia and treated with EPCs or EC-pEPCs at P7, and then angiogenesis and myelination together with cognitive outcome were evaluated at the 6th week.

**Results:**

In vitro, EPCs enhanced endothelial function and decreased OPC apoptosis. Meanwhile, it was confirmed that OGD-EC-CM induced an increase of CXCL12 in EPCs, and CXCL12-CXCR4 axis is a key signaling since CXCR4 knockdown alleviated the anti-apoptosis effect of EPCs on OPCs. In vivo, the number of EPCs and CXCL12 protein level markedly increased in the WMI rats. Compared to the EPCs, EC-pEPCs significantly decreased OPC apoptosis, increased vascular density and myelination in the corpus callosum, and improved learning and memory deficits in the neonatal rat WMI model.

**Conclusions:**

EC-pEPCs more effectively promote oligovascular remodeling and myelination via CXCL12-CXCR4 axis in the neonatal rat WMI model.

**Supplementary Information:**

The online version contains supplementary material available at 10.1186/s13287-021-02157-4.

## Introduction

Perinatal white matter damage (WMI) is the most common type of brain damage in neonatal infants, which usually causes cerebral palsy and various neurobehavioral disorders. Blood-brain barrier (BBB) disruption, oligodendrocyte precursor cell (OPC) lose, and differentiation failure caused by ischemia or neuroinflammation finally lead to dysmyelination or hypomyelination [1, 2]. Unfortunately, the effective clinical therapeutic strategies aiming for perinatal WMI are still being explored so far.

Recently, “oligovascular niche” attracted much attention due to the factor-coupling between OPCs and endothelial cells (ECs) plays important role in oligogenesis, angiogenesis, and neurogenesis [3–5]. It has been reported that EC injury caused brain-derived neurotrophic factor and fibroblast growth factor hypo-secretion and matrix metalloproteinase-9 hyper-secretion; thus, “oligovascular niche” was disrupted and then leads to OPC damage and WMI [6, 7]. Also, selective microvascular injury of white matter triggers OPC maturation arrest and demyelination in the cases of Alzheimer’s disease pathology [8]. Our recent study found although there was a transient OPC proliferation and angiogenesis in the acute phase of the neonatal rat ischemia model, serious ECs and basement membrane damage may be a major deleterious effect on myelination in the recovery stage, prompting that oligovascular repair is a strategy for ischemia-induced WMI [9].

Several studies have demonstrated that endothelial progenitor cells (EPCs) contribute to neurorepair by paracrine or cell-based effects in the adult brain [10]. Rosell et al. found that administration of EPCs or soluble factors collected from EPCs significantly increased angiogensis; however, only EPCs itself significantly increased the thickness of corpus callosum in a mouse model of ischemic stroke [11, 12], suggesting the requirement of cell-based interaction in addition to the effects of soluble factors in white matter repair. Interestingly, Maki et al. recently reported that mouse EPC secretome ameliorate white matter damage in a mouse model of cerebral prolonged hypoperfusion by boosting oligovascular remodeling [13]. Unfortunately, previous studies mostly focused on adult WMI and remyelination, and the role of human EPCs in the neonatal rat model of WMI still remains to be assessed.

Proteomic profile of mouse EPC secretome identified 38 proteins including strong stromal-derived factor-SDF-1 (also called C-X-C motif chemokine 12, CXCL12) signal (signal intensity = 159 ± 56.7) [13]. CXCL12 has been extensively investigated and considered to play a key role in angiogenesis, inflammation, and pathological pain by binding to its receptors (CXCRs), such as CXCR4 or CXCR7 [14, 15]. Several studies also reported that CXCL12-CXCR4/CXCR7 axis involved in OPC migration, proliferation, differentiation, and remyelination in the WMI animal model [16–19]. We ask whether the level CXCL12 derived from EPCs was altered under pathological condition since EPCs were accumulated in the damaged area following brain injury. If so, could conditioned medium from hypoxic endothelial cells preconditioned EPCs (EC-pEPCs) promote the ability of EPCs in augmenting oligovascular remodeling and remyelination? In addition, whether EC-pEPCs decrease OPC apoptosis and what are the possible mechanisms?

In this study, we used co-culture system in vitro and neonatal rat model of WMI induced by ischemia-hypoxia in vivo to answer the above questions. For the first time, we demonstrate that transplanted EC-pEPCs more effectively promote oligovascular remodeling and myelination via CXCL12-CXCR4 axis in the neonatal rat WMI model.

## Methods

### Primary culture of OPCs

The OPC primary culture was carried out as our previous study [20]. Briefly, Sprague Dawley (SD) rat (postnatal 1~2 days, provided by Laboratory Animal Center, Xuzhou Medical University) cortical cells were cultured in DMEM/F12 medium (Hyclone, USA) containing 10% fetal bovine serum (FBS) and 0.1% penicillin/streptomycin. Seven days later, the culture flask was shaken on a shaker at 37 °C for 1 h and then the supernatant was discarded. Fresh medium was added to the culture flask, and OPCs were separated after shaking the flask for 18 h on the shaker. Cells were centrifuged for 5 min at 600×*g* and then re-suspended in DMEM/F12 medium (HyClone, USA) supplemented with 10 ng/ml recombinant human basic fibroblast growth factor (bFGF), 10 ng/ml recombinant human platelet-derived growth factor-AA (PDGF-AA), and 2% B27 (all from Gibco, USA). Cells were seeded into a 6-well plate at a density of 1 × 10^5^ cells/ml, and the medium was changed every 2 days.

### Primary culture of human EPCs

Human EPCs were isolated from the umbilical cord blood provided by Affiliated Hospital of Xuzhou Medical University. Informed written consent for using human umbilical cord blood was obtained from the participant. Briefly, the fresh umbilical cord blood was taken and blended with 0.01 mol/l phosphate-buffered saline (PBS). The mixture was carefully and evenly poured over the Lymphocyte Separation Medium (LSM, Corning, USA). After centrifugation for 30 min at 1800*g*, the interphase (white layer) between the plasma and the separation solution was carefully extracted, washed, and spun in PBS for 10 min at 600*g* twice. The pellet was re-suspended and poured over the LSM and centrifugated at 1200*g* for 30 min. The interphase was extracted and washed with PBS. After centrifugation, the pellet was re-suspended with endothelial cell growth medium-2 (EGM-2MV) BulletKit (LONZA, Switzerland) and the cells were placed into a 25-cm^2^ culture bottle. The medium was changed every 2~3 days.

### HBMEC culture and treatment

Human brain microvascular endothelial cells (HBMECs/ECs) were purchased from ScienCell Research Laboratories, Inc. and cultured in EGM-2MV according to our lab’s protocol [21]. To investigate the influence of OGD on ECs, the cells were divided into control, OGD 3 h, 6 h, and 9 h groups. When the ECs are 60~70% confluency in culture dishes, EGM-2MV was replaced by sugar-free DMEM (HyClone, USA) and the cells were subjected to 0, 3, 6, or 9 h of hypoxia in an incubator containing 1% oxygen and 95% nitrogen and then reoxygenated for 24 h.

Fluorescence-activated cell sorting (FACS) was used to detect apoptosis of ECs. Briefly, the cells were digested and centrifugated at 1000*g* for 5 min. After washing and centrifugation twice, the pellets were re-suspended with 300 ml binding buffer, then incubated for 15 min with 5 μl Annexin V-PE and 5 μl 7-AAD (BD-pharmingen, USA). Flow cytometry (FACS Canto II, Becton-Dickinson, USA) and FlowJo software were used for apoptosis analysis and data processing.

### Conditioned medium preparation

To produce the CM, the medium was collected and concentrated 20 times using amicon ultra-15 centrifugal filter units with a 10-kDa molecular weight cut-off membrane (Millipore) by centrifugation (4000*g*, 15 min), then the medium was sterilized on 0.22-mm filters (Millipore) and stored at − 80 °C until use. OPCs were incubated in an OPC culture medium plus 20% (v/v) of 20-fold concentrated CM for apoptosis analysis.

### Detection of the influence of EPCs on HBMECs

#### Cell apoptosis test

To observe the effect of EPCs on EC viability, ECs were divided into three groups: normal control (Ctrl), ECs were exposed to OGD for 6 h and then reoxygenated for 24 h (OGD-EC), and ECs were exposed to OGD for 6 h and then were co-cultured with EPCs for 24 h (OGD-EC+EPCs). Cell apoptosis was detected by FACS.

#### Cell migration assay

To investigate the effect of EPCs on the invasion ability of OGD-ECs, ECs were seeded into the transwell chamber, which then were inserted into a 24-well plate. EPCs were cultured in another 24-well plate. After OGD for 6 h, the transwell chamber was removed and placed into the 24-well plate grown with EPCs. Meanwhile, the medium derived from OGD-EC was collected, concentrated, and used to incubate the EPCs. ECs and EPCs were co-cultured for 4 h, the migration ability of ECs was examined by crystal violet staining.

#### Tube formation assay

EPCs were seeded into the transwell chamber, which then were inserted into a 24-well plate. After ECs were exposed to OGD for 6 h, the chamber was moved to the plate grown with OGD-ECs. The OGD-ECs and EPCs were co-cultured for 24 h at 37 °C in a humidified atmosphere with 5% CO_2_, then the ECs were digested and seeded into Matrigel (Corning Inc., USA)-treated 24-well plate. Tube formation was observed with microscopy (Olympus, TKY, Japan), and the images were analyzed by ImageJ software.

#### Cellular immunofluorescence staining

Cells were fixed at room temperature for 15 min with 4% paraformaldehyde (PFA) and rinsed with 0.01 mol/l PBS. After blocking non-specific antigens with 5% BSA, OPCs were treated with mouse monoclonal anti-A2B5 antibody (1:200, Sigma, USA) and ECs and EPCs were incubated with rabbit anti-CD31 antibody (1:100, Abcam, USA) and rabbit anti-CD133 antibody (1:100, Abcam, USA) overnight at 4 °C, respectively. After washing with PBS, Dylight-488- or Dylight-649-conjugated goat anti-mouse IgG (H + L) secondary antibodies (1:500, Abbkine, USA) were added at room temperature for 2 h. The nucleus was stained with 4′,6-diamidino-2-phenylindole (DAPI, 1:200, Beyotime, China) for 10 min. The images were taken under an Olympus Bx60 fluorescence microscope (Olympus, TKY, Japan).

#### OPC processing and RNA interference

To investigate the influence of OGD-ECs and EPCs on OPCs, OPCs were divided into the normal group (DMEM/F12), CM derived from EC-treated group (EC-CM), CM derived from OGD-EC-treated group (OGD-EC-CM), and CM derived from EPC and OGD-EC co-culture-treated group (OGD-EC+EPCs-CM). OPCs were cultured for 24 h, and cell apoptosis was detected by terminal deoxynucleotidyl transferase dUTP nick end labeling (TUNEL) staining and FACS. The expression of CXCR4 and CXCR7 was analyzed by western blotting.

To examine whether OGD-EC+EPCs-CM alleviates OPC apoptosis through CXCL12 and its receptors, RNA interference was used to knock down the level of CXCR4 and CXCR7 in OPCs. Briefly, 60~70% confluent OPCs were transfected with CXCR4 or CXCR7 small interfering RNAs (siRNA) (Santa Cruz Biotechnology, USA) duplexes for 6 h using Lipofectamine 3000 (Invitrogen, Carlsbad, CA, USA) according to the manufacturer’s instructions. After incubation with the CM for another 24 h, the cells were harvested for FACS analysis.

#### TUNEL staining

OPCs grown in 48-well plates were fixed at room temperature for 15 min with 4% PFA, then were incubated in 0.3% Triton X-100 and 5% BSA at 37 °C for 30 min. After washing the cells with PBS 2~3 times, TUNEL reagent (KeyGen, China) was added to incubate the cells for 1 h at 37 °C. After rinsing the cells with PBS several times, DAPI (Beyotime Biotechnology, China) was added to each well at room temperature for 3~5 min. The photographs were taken under an Olympus Bx60 fluorescence microscope.

#### Enzyme-linked immunosorbent assay (ELISA)

To test the level of CXCL12 derived from ECs, ECs were divided into the normal control group (Ctrl-EC), OGD 6 h and then reoxygenation 24 h group (OGD-EC), EPCs co-cultured with OGD-ECs group (OGD-EC+EPCs), and EPCs co-cultured with normal ECs group (Ctrl-EC+EPCs). The medium was collected and centrifuged at 800*g* for 5 min to remove debris, and the concentration of CXCL12 in the supernatant was measured by ELISA kit (R&D Systems) according to the manufacturer’s instructions. The CXCL12 at the level of ECs was detected by PCR and western blotting.

To further investigate whether OGD-ECs influenced the secretion of CXCL12 from EPCs, EPCs were infected with CXCL12 shRNA or control lentivirus (Lentiviral Particles sc39367-v, Santa Cruz, USA) for 48 h, then were co-cultured with OGD-ECs for another 24 h. The medium was collected and CXCL12 was detected by ELISA kit.

#### Western blotting

OPCs were dissolved with RIPA splitting buffer (1% deoxycholic acid, 1% Triton X-100, and 0.1% NaN_3_) containing 10-μm-benzosulfonyl fluoride and phosphatase inhibitors. The protein concentration was determined by bicondylic acid. The protein lysate was separated by 8% or 10% twelve alkyl sulfate polyacrylamide gel electrophoresis (SDS-PAGE) and transferred to the nitrocellulose filter membrane. The membrane was incubated with 5% defatted dry milk buffer (dissolved in washing solution) for 1 h and then incubated with mouse anti-CXCR4 (1:200, Santa Cruz, USA) and mouse anti-CXCR7 (1:1000, Abcam, Cambridge, UK) primary antibodies at 4 °C overnight. After washing with PBS, the membrane was incubated with goat anti-rabbit or goat anti-mouse (1:5000, LI-COR, USA) in a shaker for 2 h at room temperature. Finally, Odyssey infrared imager (AGT, SFO, USA) was used to scan the imprinting, and ImageJ software was used to quantitatively analyze.

#### Quantitative real-time polymerase chain reaction (qRT-PCR) analysis

The ribonucleic acid (RNA) of ECs was extracted using the cold Trizol according to the manufacturer’s instructions. The primer sequences of human CXCL12 (F 5′-TGCCAGAGCCAACGTCAAG, R 5′-CAGCCGGGCTACAATCTGAA) and human GAPDH (F 5′-GCAAATTCCATGGCACCGT, R 5′-TCGCCCCACTTGATTTTGG) were used. Total RNA was then reverse transcribed into cDNA using the RevertAid first strand cDNA reverse synthesis kit (Takara). The qRT-PCR reaction was performed using a Light Cycler 480 SYBR green I Master Mix, and the thermocycling conditions were as follows: 95 °C for 10 min warm boot, 40 cycles 95 °C for 15 s and 60 °C for 45 s, and then 72 °C for 30 s. Relative gene expression was calculated using the 2^-△△ct^ method.

#### Neonatal rat ischemia-hypoxia model and treatment

SD rat pups (P3) obtained from the Center of Experimental Animals of Xuzhou Medical University were randomly divided into the normal control group, the sham group, the ischemia-hypoxia (HI) with vehicle-treated group, HI with EPC graft group, HI with EC-pEPCs graft group, and EC-pEPCs graft with AMD3100-treated group. HI was produced as our previously described [9, 22]. Briefly, the P3 rat pups were anesthetized by gaseous anesthesia with isoflurane and O_2_. The right common carotid artery was carefully isolated from the surrounding tissue and ligated. Then, the wound was sutured, and the pups were allowed to recover for 2 h before exposing to 8% O_2_ (92% N_2_ saturation) at 37 °C for 90 min in a humidified chamber. The sham group underwent the same procedures without ligation of the carotid artery. The pups were exposed to room air after hypoxia.

For transplantation, the HI pups (P3 + 4, *n* = 10 for each group) were anesthetized with 2.5% isoflurane (Shandong Keyuan Pharmaceutical Co., Shandong Province, China) in a mixture of air and 100% oxygen (1:1) and positioned in a stereotaxic apparatus. According to the coordinate (Bregma: AP − 1.0 mm, ML 0.5 mm, and DV 2.0 mm), a 26-gauge needle attached to a 5-μl Hamilton syringe was lowered into the right corpus callosum and 2 μl EPCs or EC-pEPCs (1 × 10^5^ cells/μl Hank’s Balanced Salt Solution, HBSS) or vehicle was released at a rate of 0.2 μl/min, after which the needle was left in place for 10 min to ensure complete diffusion. After operations were finished, the pups were placed on a warm pad carefully for 30 min and then returned to their mother rats.

For the immunosuppression, transplanted pups received tacrolimus (TAC, 4 mg/kg/day) and sirolimus (SIR, 3 mg/kg/day; Sigma-Aldrich) once daily by intraperitoneal injection for 3 weeks, then by drinking water (both 100 μg/ml) till sacrifice. For observing the influence of CXCR4 antagonist on transplanted EC-pEPCs, AMD3100 (5 μg/kg body weight, 10 μg/ml, Selleck, USA) was intraperitoneally injected once per day for 2 weeks. All procedures in the experiment were consistent with Chinese legislation on the use and care of laboratory animals and were approved by Xuzhou Medical University Committee for animal experiments.

#### Morris water maze (MWM) test

MWM test was performed during the sixth week after transplantation as previously described (*n* = 8 for each group) [9, 22]. The experimental apparatus consisted of a circular water tank (100 cm in diameter, 35 cm in height), containing water (23 ± 1 °C) to a depth of 15.5 cm, which was rendered opaque by adding black nontoxic carbon ink. A platform (4.5 cm in diameter, 14.5 cm in height) was submerged 1 cm below the water surface and placed at the midpoint of one quadrant. The pool was located in a test room that contained various prominent visual cues. Each rat received four training periods per day for 4 consecutive days. The latency to escape from the water maze (by finding the submerged escape platform) was calculated for each trial. On day 5, the probe test was performed by removing the platform and allowing each rat to swim freely for 60 s. The time that rats spent swimming in the target quadrant (where the platform was located during hidden platform training) and in the three nontarget quadrants (right, left, and opposite quadrants) was measured, respectively. For the probe trials, the number of times each rat crossed over the platform site was also measured and calculated. All data were recorded with a computerized video system.

#### Tissue preparation

Briefly, rats (*n* = 4 for each group) were perfused intracardially with PBS followed by 4% cold PFA at the seventh week after transplant. The brains were post-fixed in 4% PFA for 6 h, incubated in 30% sucrose buffer for 48 h at 4 °C, and then were embedded in Optimal Cutting Temperature medium (Leica Microsystems, Nussloch, Germany) for cryosection. Serial coronal sections (20 μm) were cut from the bregma anterior-posterior coordinates + 1.0 to − 0.2 for immunofluorescence and TUNEL staining.

#### TUNEL and immunofluorescence staining

For immunofluorescence, the brain sections were blocked with 10% goat serum and/or 0.3% Triton X-100 in 0.01 mol/l PBS for 40 min at 37 °C, followed by incubation with primary antibodies for MBP (1:1000, rabbit IgG, Abcam, Cambridge, UK) and platelet-derived growth factor receptor α (PDGFR-α, 1:1000, rabbit IgG, Abcam, Cambridge, UK), Claudin-5 (1:1000, rabbit IgG, Sigma), CD133 (rabbit IgG, 1:100, Abcam, USA), human CD31 (1:100, rabbit IgG, Sino Biology), and vWF (1:200, mouse IgG, Abcam, USA). After washing with PBS, the sections were incubated with the goat anti-mouse IgG (H + L) Alexa Fluor®488 or 555-conjugated or goat anti-rabbit IgG (H + L) Alexa Fluor®488 or 555 (Invitrogen, Eugene, OR, USA) secondary antibodies for 1 h. The specificity of the staining was assessed by omitting the primary antibody. After washing with PBS, TUNEL reaction fluid was added to the sections incubated with anti-PDGFRα antibody for 1 h at 37 °C. Finally, the sections were incubated with DAPI for 10 min and mounted.

The number of CD133-positive cells was counted in the corpus callosum in three sections per rat from the same levels, at every 9–12th section between bregma levels + 1.0 and − 0.2 mm. For quantitative analysis of the Claudin-5 or MBP immunofluorescence staining, integral optical density (IOD) was measured by Image-Pro Plus 6.0 software. Values (three slides for each brain) of optical density in individual cells represented the quantity of objective protein and were calculated using the following equation: Σ IOD/Σ DAPI. The apoptotic rate was calculated using the following formula: apoptosis rate = TUNEL and PDGFRα double-positive cells/total PDGFRα-positive cells per field × 100%.

#### Electron microscopy

For electron microscopic examination, Epon embedding was performed as previously described [23]. In brief, the rats (*n* = 4 for each group) were transcardially perfused with 2% glutaraldehyde (Gla) and 2.5% PFA in 0.1 mol/l PBS. Brains were quickly removed and placed on ice. The corpus callosum was dissected and placed in 3% Gla in 0.1 M cacodylate buffer (pH 7.4) at 4 °C overnight and transferred to 1% osmium tetroxide in the same buffer for 1 h at room temperature. Tissue was transversely cut into 1-mm blocks that were fixed in osmium tetroxide at 4 °C overnight, dehydrated through ascending ethanol washes, and embedded in epoxy resin. To study the remyelinated axons of the corpus callosum, serial 1-μm semi-thin sections were cut and stained with 1% toluidine blue and examined by light microscopy. To analyze myelin sheaths in the corpus callosum, 60~70-nm-thick ultra-thin sections were stained with uranyl acetate and lead citrate prior to examination by tEM (FEI Tecnai G2 T12, USA), and the image was analyzed by TEM Imaging and Analysis. For morphometric analysis, the axonal diameter (*d*) as the shortest distance across the center of the axon was measured. The axonal diameter plus the total myelin sheath thickness on both sides was defined as the fiber diameter (*D*). The *g*-ratio was calculated using the *d*/*D* ratio.

### Statistical analysis

All statistical analyses were conducted by observers blinded to the treatment. All data shown represent means ± SD from triplicate experiments performed in a parallel manner. Statistical analysis was performed with GraphPad Prism® software. Group differences in the MWM test were analyzed using a two-way analysis of variance (ANOVA). The other data were evaluated by one-way ANOVA and Student-Newman-Keuls (SNK) tests with homogeneity of variance or by Dunnett’s post hoc test with square differences. *P* < 0.05 was considered to be statistically significant.

## Results

### EPCs enhanced EC viability, migration, and tube formation

**Fig. 1:**
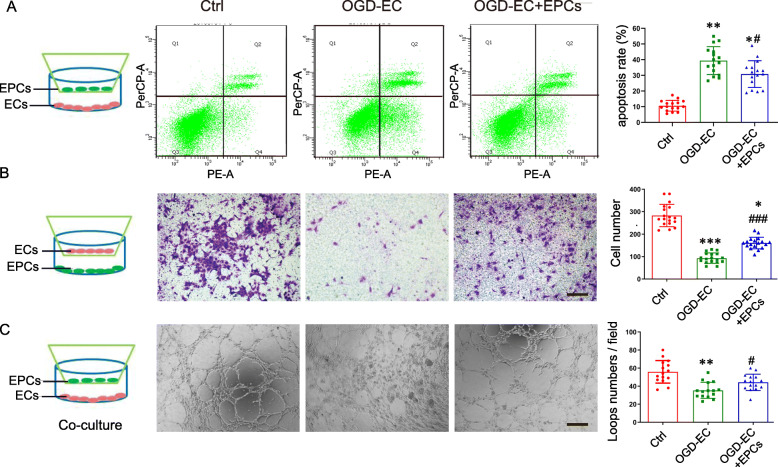
EPCs reduced OGD-induced EC apoptosis and enhanced its function. **a** FACS was used to detect apoptosis of ECs. **b** Representative images of ECs with crystal violet staining and quantitative analysis of the ECs number passed through the polycarbonate membrane. **c** Representative images show the tubes formed by ECs and the number of tubes was quantitatively analyzed. Scale bar = 200 μm in **b** and **c**. Comparing the mean ± SD (*n* = 6, triplicates per group), *P < 0.05, **P < 0.01, ***P < 0.001 vs. Ctrl group; ^#^*P* < 0.05, ^###^*P* < 0.001 vs. OGD group. Data were analyzed using Student’s *t* test

The characteristics of OPCs, HBMECs, and EPCs were identified using their specific markers (Supplemental Fig. 1A-C). FACS and CCK-8 results showed that EC apoptosis rate and viability of ECs was significantly decreased OGD 6 h or 9 h compared to OGD 3 h, but there is no significant difference between OGD 6 h and 9 h (Supplemental Fig. 2). Thus, OGD 6 h was selected for the following experiments. Compared to the OGD group, EPC treatment resulted in a significant decrease of EC apoptosis rate (Fig. 1a), enhancement of the migration (Fig. 1b), and the number of loops in tube formation assay (Fig. 1c).

### EPCs reduced OPC apoptosis induced by conditioned medium of OGD-ECs

**Fig. 2:**
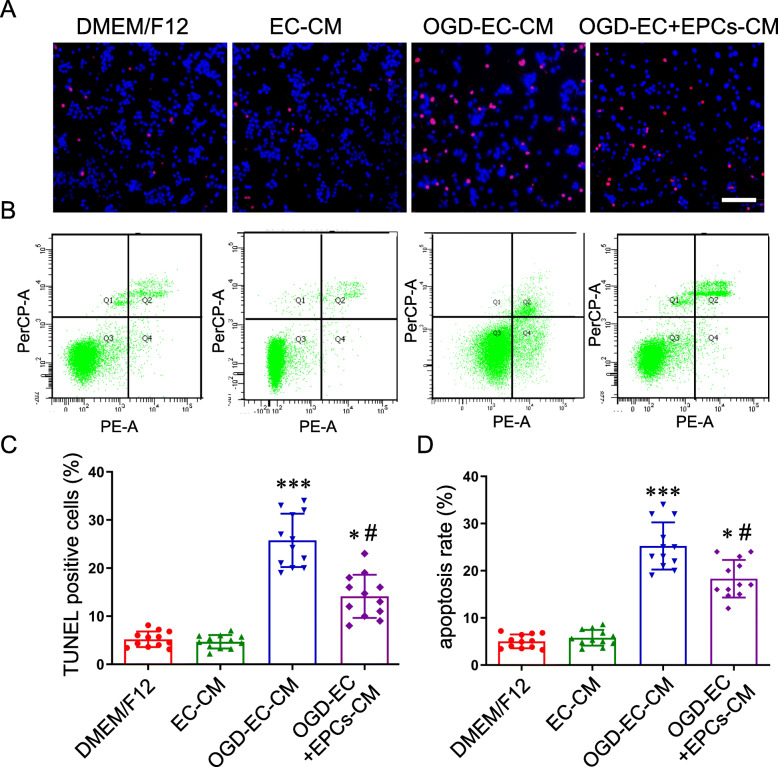
The OPC apoptosis was induced after they were incubated for 24 h with the conditioned medium (CM) from OGD-ECs. **a** Representative images of OPCs with TUNEL staining, scale bar = 200 μm. **b** OPC apoptosis induced by the CM from OGD-ECs was detected by FACS. **c** Quantitative analysis of apoptotic cell number and apoptosis rate of OPCs, respectively. Comparing the mean ± SD (*n* = 4, triplicates per group), *P < 0.05, ***P < 0.001 vs. DMEM/F12 group; ^#^*P* < 0.05 vs. OGD-EC-CM group. Data were analyzed using Student’s *t* test

We compared the influence of CM from OGD-ECs and OGD-ECs/EPCs co-culture on OPC apoptosis. The results showed that OGD-EC-CM resulted in an increase in the percentage of TUNEL-positive OPCs compared to the DMEM/F12 group. In contrast, OGD-ECs+EPCs-CM treatment markedly reduced the percentage of OPC apoptosis compared to the OGD-EC-CM group (Fig. 2a, c). Similarly, data from FACS also indicated that EPC treatment significantly decreased OPC apoptosis induced by OGD-EC-CM (Fig. 2b, d).

### EPC-derived CXCL12 alleviated OPC apoptosis through CXCR4, but not CXCR7

**Fig. 3:**
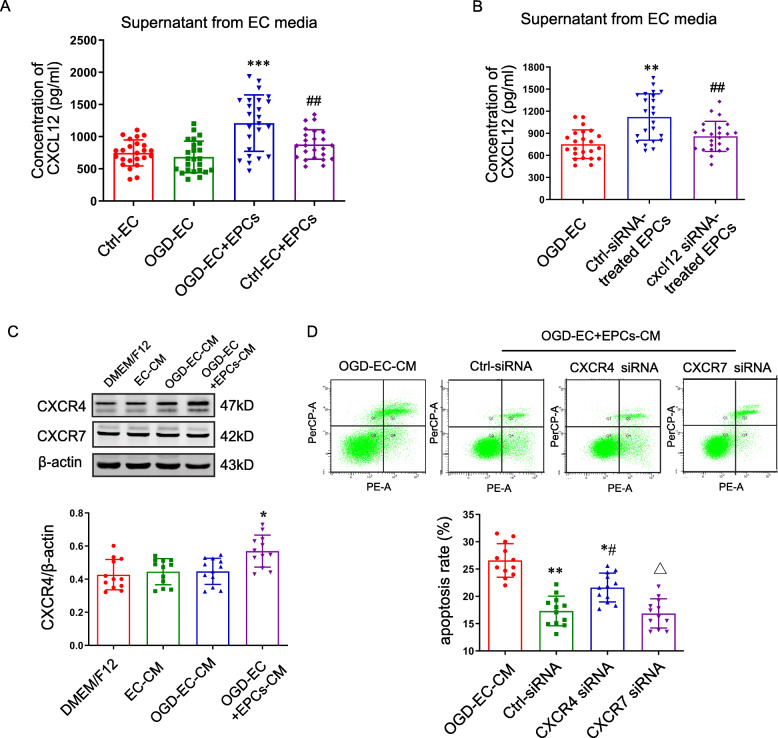
Conditioned medium derived from EPCs and OGD-ECs co-culture alleviates OPC apoptosis through CXCL12-CXCR4. **a**, **b** The concentration of CXCL12 in the supernatant of ECs in each group was detected by ELISA (*n* = 9, triplicates per group). **P < 0.01 vs. OGD-EC group; ^##^*P* < 0.01 vs. OGD-EC+EPCs or control-siRNA group. **c** The expression of CXCR4 and CXCR7 in OPCs was detected and analyzed after incubated for 24 with the CM (*n* = 4, triplicates per group). **P < 0.01 vs. OGD-EC-CM group. **d** The apoptosis of OPCs was increased with CXCR4 silence (*n* = 4, triplicates per group). *P < 0.05, **P < 0.01, ***P < 0.001 vs. EC-CM group; ^#^*P* < 0.05 vs. control-siRNA group; ^△^*P* < 0.05 vs. CXCR4-siRNA group. Data were analyzed using Student’s *t* test

Given that EPCs have the ability to secrete CXCL12, we next investigated whether EPC-derived CXCL12 was changed under pathologic conditions. There was no obvious difference in the CXCL12 level among the control ECs, OGD-ECs, and Ctrl-EC+EPCs groups. Interestingly, CXCL12 in the medium of OGD-EC+EPCs increased remarkably compared to the OGD-ECs group (Fig. 3a). However, the expression of CXCL12 mRNA and protein in each group of ECs were similar (Supplemental Fig. 3A-B). Therefore, we supposed that the increased CXCL12 be produced from EPCs, but not ECs. Consistent with our hypothesis, we demonstrated that CXCL12 was produced from EPCs by cxcl12 knockdown and OGD-ECs triggered the production and secretion of CXCL12 in EPCs. The CXCL12 in the supernatant of cxcl12-silencing EPCs was sharply decreased compared to the vector group (Fig. 3b).

Whether the CM from OGD-EC+EPCs alleviates OPC apoptosis remains elusive. We further detected the expression of CXCL12 receptors CXCR4 and CXCR7 in OPCs. The expression of CXCR7 was not affected by the CM treatment; however, the expression of CXCR4 significantly increased in the OPCs treated with OGD-EC+EPCs-CM (Fig. 3c). The expression of CXCR4 and CXCR7 were obviously downregulated by cxcr4 and cxcr7 silence (Supplemental Fig. 3C-D). Compared to the control siRNA, cxcr4 but not cxcr7 knockdown significantly increased OPC apoptosis (Fig. 3d).

### EPCs and CXCL12 increased in a neonatal rat model of WMI

**Fig. 4:**
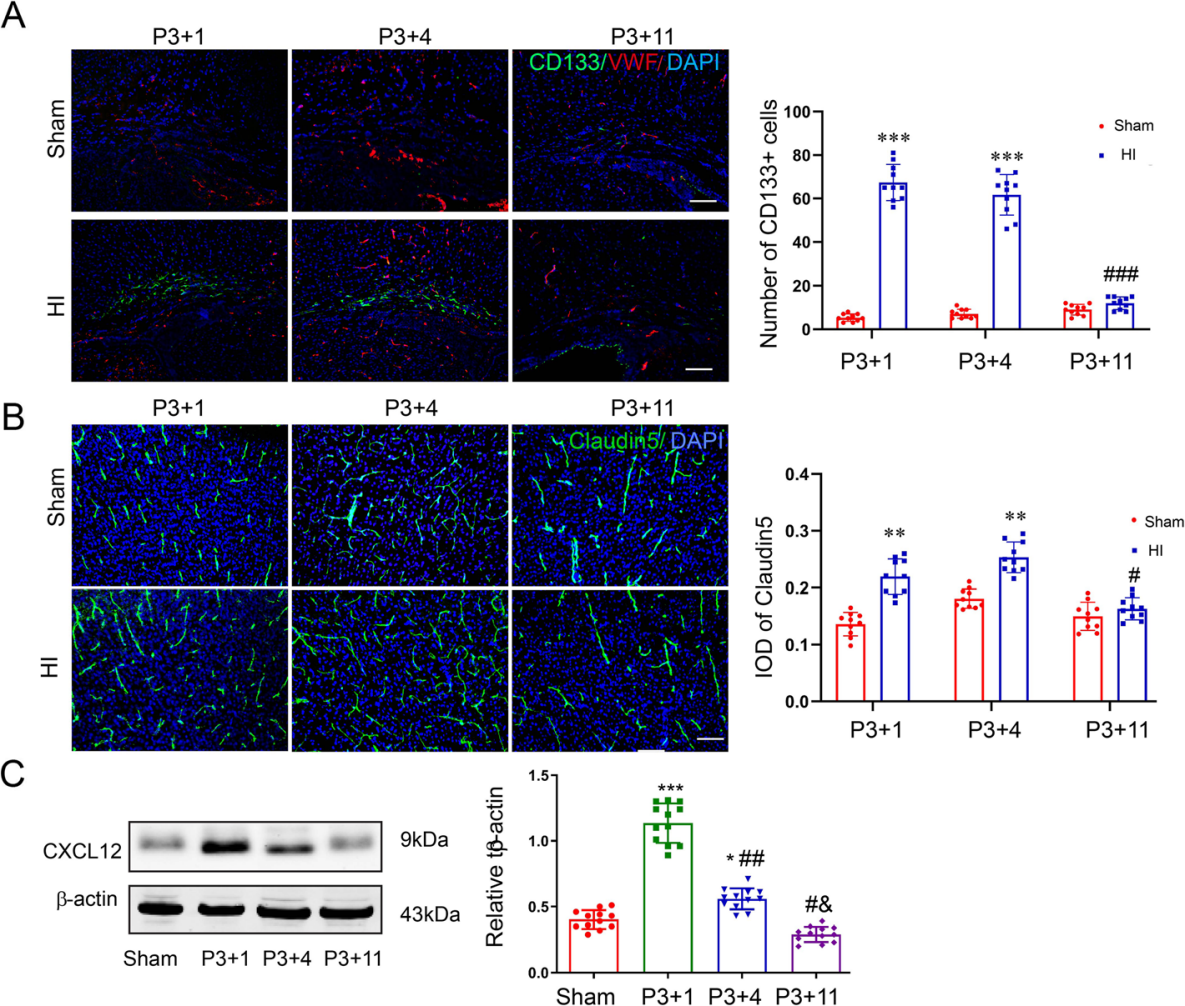
Ischemia-hypoxia leads to EPC accumulation and CXCL12 increase in the corpus callosum of neonatal rat. **a**, **b** The accumulation of EPCs and claudin5-positive vessels in the corpus callosum were observed by immunofluorescence, and the number of EPCs and the density of vessels were quantified. Scale bar = 200 μm in **a** and 50 μm in **b**. **c** CXCL12 protein level of the corpus callosum was examined by western blotting. Data were analyzed using one-way ANOVA and shown as mean ± SD (*n* = 4, triplicates per group). *P < 0.05, **P < 0.01, **P < 0.001 vs. Ctrl group; ^#^*P* < 0.05, ^##^*P* < 0.01, ^###^*P* < 0.001 vs. P3+1 group; ^Δ^*P* < 0.05 vs. P3+4 group. Data were analyzed using Student’s *t* test

Our previous study has demonstrated that both oligogenesis and angiogenesis were increased at the early stage, but decreased at the recovery stage in HI-induced WMI neonatal mouse model [9]. We asked whether EPCs are involved in oligogenesis and angiogenesis due to their neuroprotective function. Interestingly, in this study, we found that EPC number was significantly increased in the corpus callosum in the model rat at P4 and P7, but lowered to normal level at P14 (Fig. 4a). The mean optical density of claudin5-positive vessels in the model rat was higher than that in the sham rat at the early stage of HI (Fig. 4b). In addition, HI also leads to an increase in CXCL12 protein level (Fig. 4c).

### EC-pEPCs graft more effectively promoted oligovascular remodeling and improved cognitive function of WMI rat

**Fig. 5:**
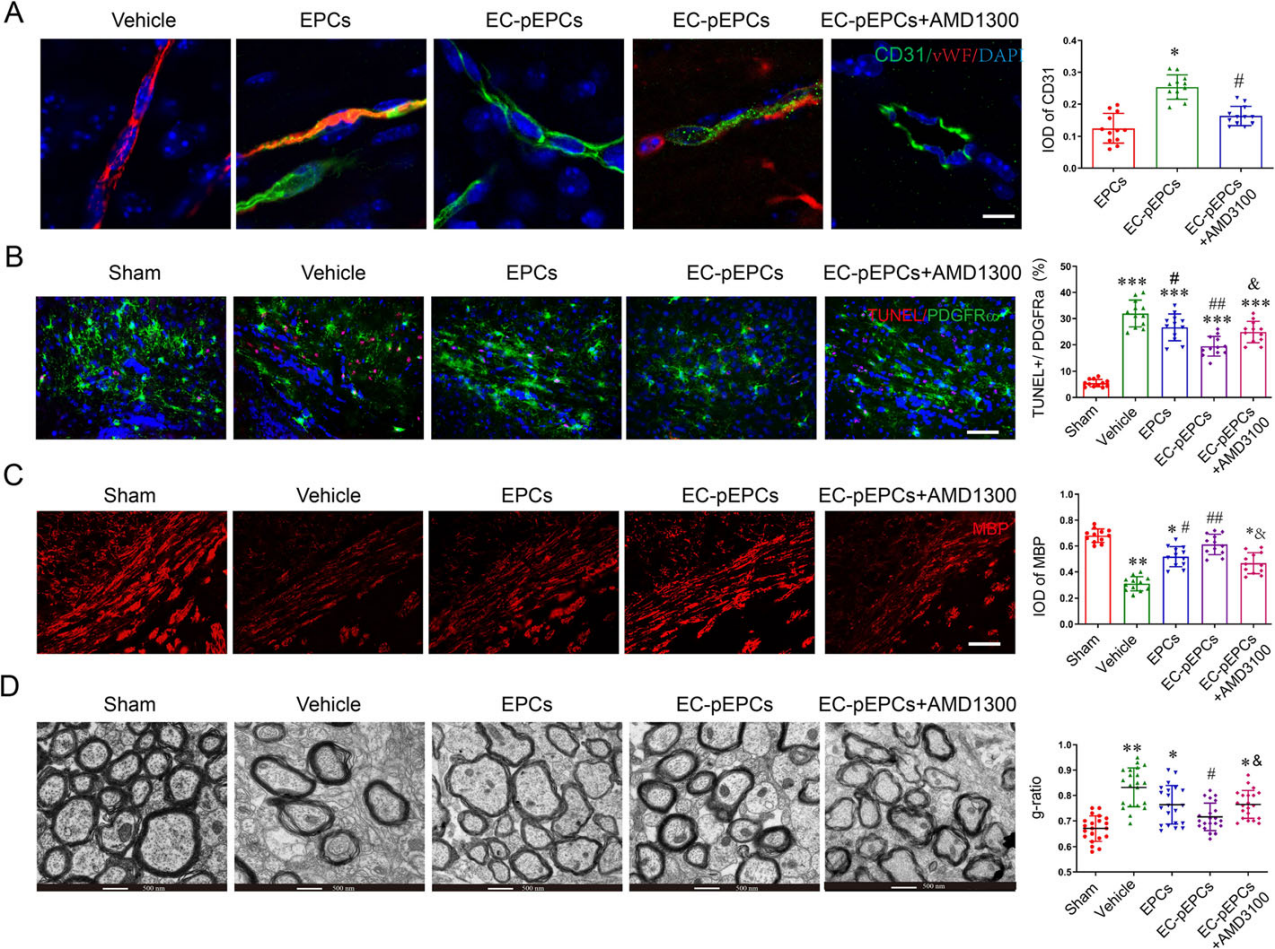
Transplantation of EPCs promoted oligovascular remodeling in WMI rat. **a** Immunofluorescent staining showed EPC-derived CD31-positive ECs (green) integrated into the host brain and formed vascular-like structure, scale bar = 5 μm. The IOD of CD31 was quantified. **b** HI-induced OPC apoptosis was detected by PDGFRα immunofluorescent and TUNEL staining, scale bar = 20 μm. The percent of TUNEL and PDGFRα double-positive cells was analyzed. **c** MBP immunofluorescent staining and quantitative analysis were conducted for myelination evaluation, scale bar = 20 μm. **d** Representative electron microscopic images of the corpus callosum were shown. The *g-*ratio was calculated in each group. All values are expressed as the mean ± SD (*n* = 4 for each group). *P < 0.05, **P < 0.01, ***P < 0.001 vs. sham group; ^*#*^*P* < 0.05, ^*##*^*P* < 0.01 vs. vehicle group; ^Δ^*P* < 0.05 vs. EC-pEPCs group. Data were analyzed using Student’s *t* test

**Fig. 6:**
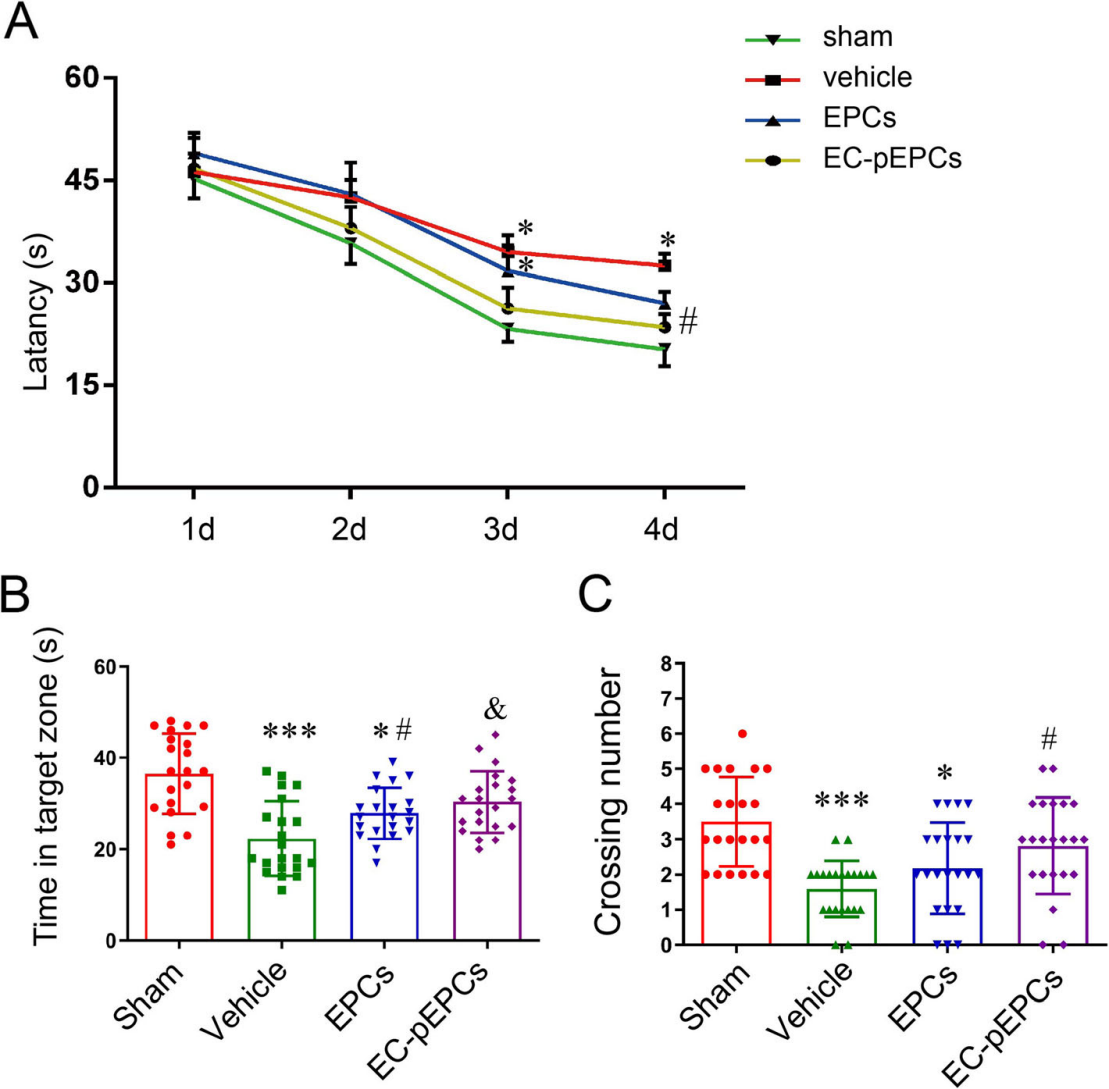
EC-pEPCs graft more effectively reversed cognitive deficits in the WMI rat. **a** Mean latency in the MWM hidden platform test was analyzed by two-way ANOVA. **b**, **c** Comparison of the time spent in the target quadrant and the crossing number over the exact location of the former platform on the fifth day. Data were analyzed using Student’s *t* test (*n* = 8 for each group). *P < 0.05, ***P < 0.001 vs. sham group; ^#^*P* < 0.05 vs. vehicle group, ^Δ^*P* < 0.05 vs. EPCs group

We next investigated whether OGD-EC-CM preconditioned EPCs (EC-pEPCs) more effectively promote oligovascular remodeling via CXCR4 signal in vivo. Vascular density formed by grafted EC-pEPCs in the corpus callosum was enhanced compared to the normal EPC transplant rat, but AMD3100 treatment significantly decreased the vascular density formed by grafted EC-pEPCs (CD31 positive in Fig. 5a). The number of OPC apoptosis (TUNEL^+^/PDGFRα^+^) was also decreased by EPC graft, although there was no obvious difference between EPCs and EC-pEPCs groups, AMD3100 treatment significantly increased the OPC apoptosis in the rats treated with EC-pEPCs (Fig. 5b). Similarly, the rats grafted with EC-pEPCs showed more stronger fluorescent density of MBP (Fig. 5c) and significantly decreased *g-*ratio value (Fig. 5d) than those in EPC-grafted rats, but they were significantly reversed by AMD3100 treatment (Fig. 5c, d). Finally, cognitive function was measured by MWM. EC-pEPCs transplantation significantly decreased the escape latency of rats with WMI on the third and fourth day (Fig. 6a). The rats treated with EC-pEPCs showed a significant increase in the swimming time and crossing number in the target quadrant compared to the EPCs group (Fig. 6b, c).

## Discussion

Recently, the importance of OPC and EC crosstalk and paracrine of cytokines in mylin sheath development has been confirmed [3–5]. Thus, oligovascular remodeling is considered to be an effective strategy to improve myelin injury and neurological deficits after WMI [13]. In the current study, we demonstrate that EC injury induced an increase of CXCL12 in EPCs, which enhanced EC function and decreased OPC apoptosis in vitro by CXCL12-CXCR4 axis. Then, our findings further support the oligovascular remodeling and myelination function of grafted EC-pEPCs in a neonatal rat model of WMI (Supplemental Fig. 4).

It is similar to the neurovascular niche in cerebral gray matter; emerging data suggest an oligovascular niche also exists in the white matter, wherein EC and OPC interact to sustain microenviromental homeostasis in mammalian brain [24]. Arai and Lo advocated it conditioned media from human cerebral ECs contributed to OPC proliferation and attenuated OPC injury by activating Src and Akt signaling [3, 4]. Then, Tsai et al. reported that physical interactions with ECs are required for OPC migration and differentiation [25]. Afterwards, several studies demonstrated that destruction of oligovascular niche triggered CNS inflammation, blocked oligodendroglial differentiation, caused cerebral small vessel disease vulnerability, and aggravated white matter damage [26–29]. Here, we reported conditioned media from OGD-ECs caused OPC apoptosis in vitro. Hence, we next considered what strategies should be adopted to promote oligovascular repair and attenuate OPC apoptosis under the conditions of oligovascular niche destruction.

It has been reported that EPCs in systemic circulation can be activated and migrated into the injured region and incorporated into neovessels by cell replacement in adult ischemia models [30–32]. EPC decrease in systemic circulation will increase the risk of WMI and cognitive impairment [33]. High-mobility group box 1 (HMGB1) from reactive astrocytes may attract EPCs to promote recovery after WMI [34]. In the current study, we also detected EPC accumulation in the corpus callosum of the neonatal rat model of WMI. EPC-derived several trophic factors known that CXCL12, VEGF, angiogenin, or MMPs could be responsible for maintaining the oligovascular niche in the normal and injured brain [13, 35]. Of which, angiogenin has shown the ability in ameliorating WMI in a mouse model of cerebral prolonged hypoperfusion [13]. Here, we found that CXCL12 was increased in the WMI rats and supposed that it was released by the accumulated EPCs under pathologic conditions, which may contribute to oligovascular remodeling.

In vitro, EPCs decreased the apoptosis of OGD-ECs and enhanced their function. Previous studies suggested that EPCs promote angiogenesis and enhance myelin thickness in the ischemic stroke mice by secreting BDNF and bFGF. Wang group recently reported that the CM derived from cxcl12-overexpressed EPCs augmented remyelination properties of OPCs in vitro [17]. Transplantion of cxcl12-overexpressing EPCs into stroke mice brought an increase of blood vessel density and OPC proliferation [35], suggesting white matter repair is partly dependent on EPC replacement or secreting cytokines. Interestingly, in the present study, we detected an obvious increase of CXCL12 in EPCs caused by the stimulation of CM from OGD-ECs. Moreover, treatment with the CM from OGD-ECs and EPC co-culture decreased the OPC apoptosis. For the first time, we reported that CXCR4 was upregulated whereas CXCR7 was not altered when OPCs were exposed to the CM from OGD-ECs and EPC co-culture. Further, the data from RNAi suggest that CXCR4 is responsible for CXCL12-dependent anti-apoptosis in OPCs, but not CXCR7. In this work, we also conducted an iTRAQ-based quantitative proteomic analysis, and the data reveals that 295 proteins were downregulated and 305 proteins were upregulated in OGD-ECs compared to normal control ECs (data not shown). It was known that several factors such as IL-17, HIF-1α, E2, and FGF-2 could regulate the expression of CXCL12 [36–39], and we are performing an in-depth study for the mechanisms of CXCL12 increase in EPCs in another study.

EPCs-CM or EPC transplantation improves the white matter integrity, decreases capillary breakdown, and inhibits apoptosis after traumatic brain injury (TBI) [12, 40]. EPC transplantation leads to recovery of white matter, enhancement of neurogenesis, and reducing BBB permeability in a mouse model of stroke [41]. MSC-derived CXCL12 niche played an important role in the development of EPCs and enhanced the efficiency of EPC therapy for ischemic diseases [42]. Here, we compared the effect of EPCs and EC-pEPCs on oligovascular remodeling and myelination in a neonatal rat model of WMI. The data suggest that EC-pEPCs more effectively contribute to angiogenesis, myelination, and OPC survival, and the effect of EC-pEPCs is mediated at least partly by the CXCL12-CXCR4 axis. In addition, we also evaluated the cognitive function after EPC graft. The functional outcome of WMI rat was more obviously enhanced by EC-pEPCs treatment compared to EPC treatment using the MWM test. The effects of EPC graft on longer-term oligovascular remodeling and function recovery deserve to be further evaluated.

## Conclusions

In the present study, we demonstrate for the first time that the CM from OGD-ECs induced an increase of CXCL12 in EPCs, and the CXCL12-CXCR4 axis plays a key role in anti-apoptosis of EPCs on OPCs. The targeting of the oligovascular remodeling using EC-pEPCs represents a potentially therapeutic strategy for ischemic and hypoxic neonatal WMI.

## Supplementary Information

**Additional file 1.**

## Data Availability

The data that support the findings of this study are available from the corresponding authors upon reasonable request.

## Abbreviations

BBB: Blood-brain barrier
bFGF: Basic fibroblast growth factor
BMECs: Brain microvascular endothelial cells
BSA: Bovine serum albumin
CM: Conditioned medium
CXCL12: Chemokine C-X-C motif ligand 12
CXCR4: Chemokine (C-X-C motif) receptor 4
ECs: Endothelial cells
EGM-2MV: Microvescular Endothelial cell growth medium-2
EPCs: Endothelial progenitor cells
FACS: Fluorescence-activated cell sorting
FBS: Fetal bovine serum
OGD: Oxygen-glucose deprivation
OPCs: Oligodendrocyte precursor cells
PBS: Phosphate-buffered saline
PDGF-AA: Platelet-derived growth factor-AA
RT-PCR: Real-time polymerase chain reaction
TUNEL: Terminal deoxynucleotidyl transferase dUTP nick end labeling
WMI: White matter injury
