# Supplementary material for: Conditioned medium-preconditioned EPCs enhanced the ability in oligovascular repair in cerebral ischemia neonatal rats
Source: Stem Cell Res Ther. 2021 Feb 12;12:118. doi: 10.1186/s13287-021-02157-4 (PMC7881622; doi:10.1186/s13287-021-02157-4)
Supplement: Supplementary file 1 — Additional file 1. [file 13287_2021_2157_MOESM1_ESM.docx]

**Supplemental material**

**Supplementary Figure 1**


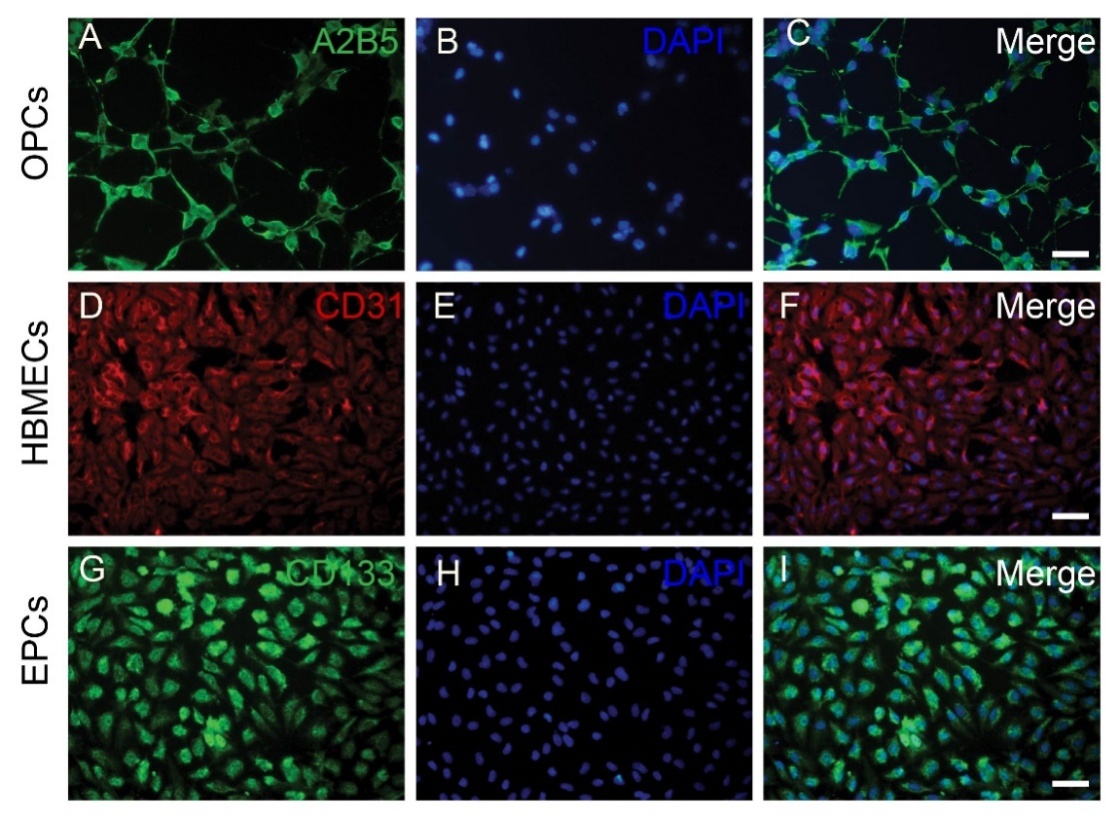


**Figure1**. OPCs, HBMECs and human EPCs were identified by A2B5 (A-C), CD31(D-F) and CD133 (G-I) immunofluorescent staining. Scale bars: 20μm.

**Supplementary Figure 2**


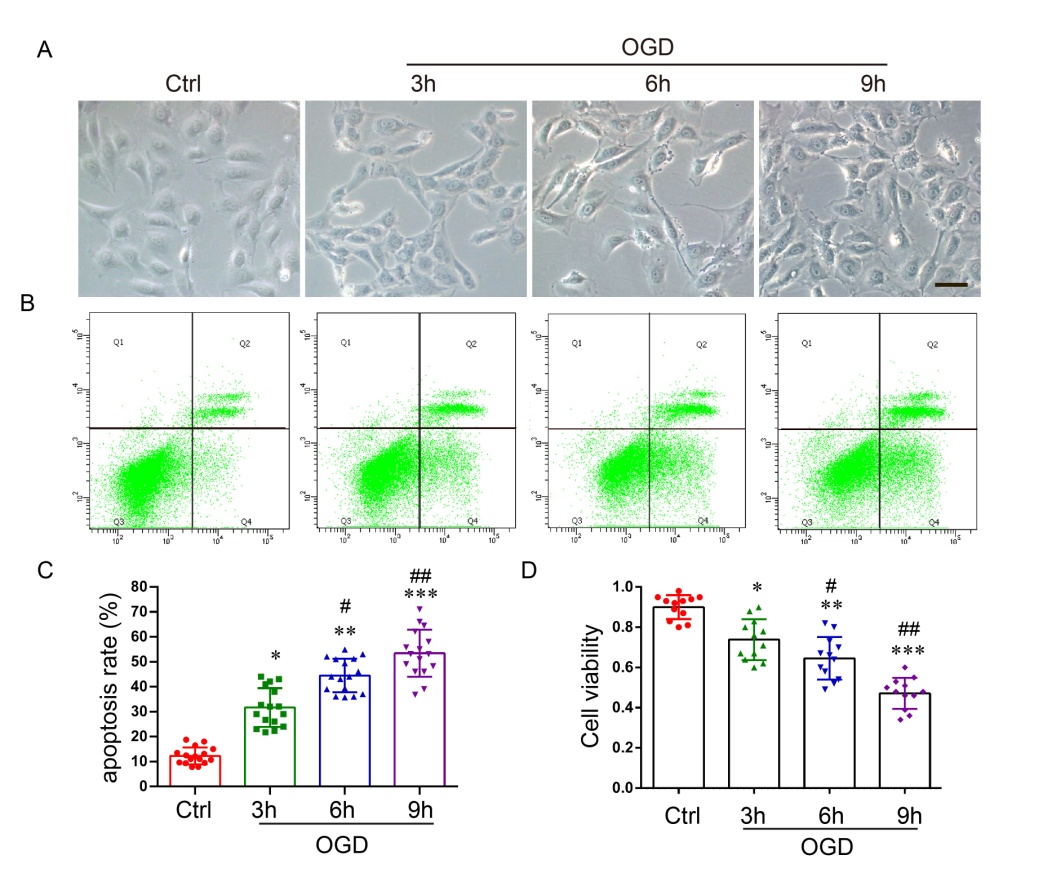


**Figure 2.**Oxygen-glucose deprivation (OGD) caused morphological changes and apoptosisof ECs. A,OGD caused morphological changes of ECs.Scale bar=50μm. **B-C,** ODG induced ECs apoptosis was detected by FACS and quantitative analyzed at different time points (n =4, triplicates per group).**D,** OGD resulted in significant reduction of ECs viability by CCK-8 analysis (n =6, triplicates per group). Data were analyzed using one-way ANOVA and shown as mean ± SD. **P*< 0.05, ***P*< 0.01, ***P*< 0.001 vs. Ctrl group; ^#^*P*< 0.05, ^##^*P*< 0.01 vs. OGD 3 h group.

**Supplementary Figure 3**


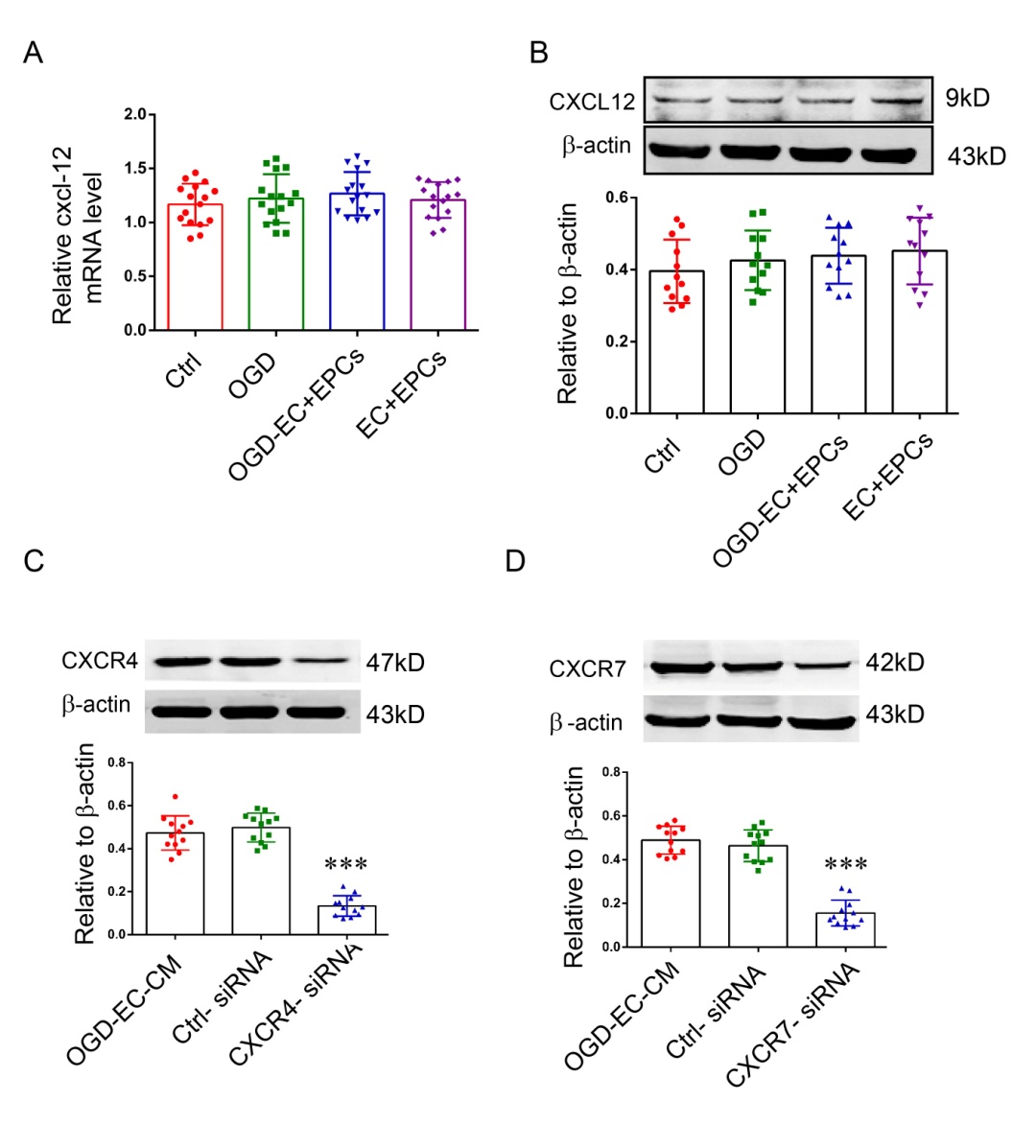


**Figure 3.** The mRNA and protein of CXCL12 of ECs were detected by RT-qPCR and western blotting. **A**, There was no statistically differencesin the CXCL12 mRNA among the groups.**B**,Quantification of CXCL12protein levels from immunoblots, and no statistically significant differences among the four groups. **C-D,** The expression of CXCR4 and CXCR7 were obviously down-regulated by cxcr4 and cxcr7 silence. Compared the mean ± SD by one-way ANOVA (n =4, triplicates per group).

**Supplementary Figure 4**





**Figure 4. A,** The transplanted EC-pEPCs (conditioned medium from hypoxic endothelial cells preconditioned EPCs) were observed by immunofluorescent stainingwiththe antibody that specifically binds to human CD133. EC-pEPCs distributed along corpus callosum. **B**, A schematic diagram was used to illustrate the effects and mechanism of EC-pEPCs on OPCs and oligovascular remodeling *in vitro* and *in vivo*.
